# Evaluation of a school based comprehensive sexuality education program among very young adolescents in rural Uganda

**DOI:** 10.1186/s12889-019-7805-y

**Published:** 2019-10-28

**Authors:** Elizabeth Kemigisha, Katharine Bruce, Olena Ivanova, Els Leye, Gily Coene, Gad N. Ruzaaza, Anna B. Ninsiima, Wendo Mlahagwa, Viola N. Nyakato, Kristien Michielsen

**Affiliations:** 1 Mbarara University of Science and Technology, P.O. Box 1410, Mbarara, Uganda; 2 International Centre for Reproductive Health, Faculty of Medicine and Health Sciences, Ghent University, Ghent, Belgium; 3 Division of Infectious Diseases and Tropical Medicine, Medical Centre of the University of Munich (LMU), 80802 Munich, Germany; 4 Centre of Expertise on Gender, Diversity and Intersectionality, Vrije universiteit Brussels, Brussels, Belgium

**Keywords:** Comprehensive sexuality education, Sexual and reproductive health, Evaluation, Very young adolescents, Uganda

## Abstract

**Background:**

Limited research has been conducted on the effectiveness of sexuality education for very young adolescents (VYAs) ages 10–14 years in Sub-Saharan Africa. Furthermore, evaluations of sexuality education programs often report outcomes of risky sexual practices, yet positive aspects of sexuality are hardly studied and rarely reported. This study evaluates the effectiveness of a Comprehensive Sexuality Education (CSE) intervention for VYAs in Uganda, analyzing both positive and negative outcome indicators.

**Methods:**

We conducted a mixed methods study, incorporating a cluster randomized trial (NCT03669913) among pupils in 33 randomly selected primary schools in Mbarara district. This was followed by a qualitative evaluation of the intervention in 4 schools that included 14 in-depth interviews and 3 focus group discussions distributed among pupils, teachers and parents. Quantitative data were analyzed using ordered logistic regression to compare differences in the change from baseline to endline between the intervention and control arms. We conducted bivariate analysis and multiple regression analysis controlling for key covariates, including age, gender, school location (rural vs urban), truancy, and orphanhood. Qualitative data were analyzed by thematic approach using ATLAS TI.

**Results:**

Between July 2016 and August 2017, 1096 pupils were recruited. Outcomes were studied among 380 pupils in the intervention arm and 484 pupils in the control arm. The proportion of pupils who ever had sex increased from 9 to 12.1% in intervention compared to 5.2 to 7.4% in the control group between baseline and endline, however the differences between groups were not statistically significant. We found greater improvements in sexual and reproductive health (SRH) knowledge among intervention schools (AOR: 2.18, 95% CI: 1.66–2.86) and no significant differences in self-esteem, body image or gender equitable norms. Qualitative evidence echoes perceived SRH knowledge acquisition, increased their perception of SRH related risks, and intentions to delay sexual intercourse to prevent unwanted pregnancy, HIV and other STIs.

**Conclusion:**

This study demonstrates that CSE can improve SRH knowledge and behavioral intentions among VYAs in Uganda. These results further emphasize the importance of initiating sexuality education before most adolescents have started engaging in sexual activity, enabling them to make informed decisions in the future.

**Trial registration:**

NCT03669913, registered retrospectively on September 13th, 2018.

## Background

Sub-Saharan Africa (SSA) has a largely youthful population whereby in 15 countries, more than half of the population is below 18 years [1]. Adolescents between 10 to 19 years make up at least 23% of the population in SSA and these face the highest burden of Sexual and Reproductive Health (SRH) risks [2–4]. It is estimated that in 2016, 777,000 births occurred among young adolescents ages 10 to 14 in developing countries, 58% of which were in Africa [5]. In Uganda, over a third of the population are adolescents [6], most of whom face great challenges related to their sexual health and wellbeing. According to Uganda’s 2016 Demographic and Health Survey (UDHS), 25% of girls begin child-bearing by the age of 19 years [6]. Adolescent pregnancy is associated with immediate health risks, including pre-term delivery, operative delivery, eclampsia and poor infant survival, as well as the long-term risks of dropping out of school and the financial burden of child-bearing [7, 8]. Furthermore, the HIV prevalence in Uganda remains high. About 1.2 million adults and 95,000 children below 15 years are living with HIV/AIDS, with a prevalence of 6.2 and 0.5% respectively [9]. Many Ugandan adolescents lack the knowledge necessary to make informed decisions about their SRH. The 2016 DHS found that only 46% of females and 45% of males aged 15–24 had comprehensive knowledge about HIV prevention [6]. These SRH issues demonstrate a complex intersection of limited resources, lacking SRH knowledge, and prohibitive cultural norms, all of which influence SRH behaviors [10, 11].

Most research and country-level data in Uganda focuses on older adolescents, aged 15 and above, while very young adolescents are often neglected in this field. Interventions usually focus on older adolescents who may already be sexually active, yet interventions that focus on younger adolescents who have not experienced sexual debut may be more effective in preparing these adolescents to make informed preventive choices. Comprehensive Sexuality Education (CSE) could be an effective and feasible approach to improve adolescent SRH knowledge and outcomes. UNESCO defines CSE as “an age-appropriate, culturally relevant approach to teaching about sexuality and relationships by providing scientifically accurate, realistic, non-judgmental information” [12]. A meta-analysis of 33 school-based CSE programs implemented in low and middle income countries showed that CSE can increase sexual health knowledge and safer sexual practices such as delayed sexual debut, increased condom use and a reduced number of sexual partners [13]. However, few such studies have been published on the Ugandan context. In the two school-based CSE evaluation studies found in Uganda, effectiveness was limited, due to challenges in implementation, lack of fidelity to the program, and poorly defined outcome indicators [14–16]*.* The majority of existing studies, in Uganda and elsewhere, focus on older adolescents, and there is a need for further research among young adolescents where such programs could be more effective [17]. Furthermore, most studies only evaluate risky or negative behaviors and attitudes. Few measure positive behavioral components related to CSE interventions such as life skills, self-esteem and gender equitable norms [18].

This paper evaluates the effectiveness of a comprehensive SRH intervention implemented in 33 primary schools for very young adolescents (VYAs) in South Western Uganda. The evaluation explored changes in knowledge and behavior, in addition to more novel measures of positive attributes of sexual health including self-esteem, body image, and gender equitable norms.

## Methods

### The intervention

The intervention was based on behavioral theories including the Theory of Planned Behavior and the Social Ecological Model to promote changes in knowledge, attitudes and practices related to sexual health [19, 20]. The intervention was developed following extensive document review of international guidance on sexuality education [12], standards for sexuality education in Europe [21] and curriculum for primary schools in Uganda for primary level 4–7 (between an average age of 9 to 12 years) [22]. The lessons employed diverse classroom activities including role playing, group discussions, dissecting case studies, individual written activities and traditional lectures. A Community Advisory Board (CAB) consisting of educational, religious and cultural leaders in Mbarara helped to provide insight into the cultural and religious appropriateness of all content delivered. There were 8 CAB members in total. The CAB met with the research team a total of 4 times (prior to the baseline survey, prior to the start of the intervention, during the course of the intervention and at the dissemination of findings). The CAB was instrumental in guiding the implementation process and recommending changes in the evaluation questionnaire and lesson content to ensure cultural appropriateness.

Eleven lessons were delivered that consisted of the following topics: puberty, relationships and emotions, decision making, self-esteem skills, reporting of physical and sexual violence, knowing one’s rights, sexually transmitted infections, HIV/AIDS and stigma, prevention of pregnancy, sexuality and gender, and sexuality and media influence. Volunteer undergraduate university students were trained in facilitation skills for each lesson prior to its delivery in schools. There were a total of 22 educators at the beginning of the intervention of which 13 were female and 9 were male. During the intervention, five educators graduated from university and were replaced with other university students. Each of the eleven lessons was delivered within 1–2 h in each of the schools to the combined upper classes of primary five, six and/or seven. However, sometimes two lessons were combined and delivered in one session. A total of 8 visits were made on a monthly basis to each of the 15 intervention schools to deliver the lessons. The delivery of these lessons was monitored by observers including teachers at each school and lecturers and post graduate students from Mbarara University. Throughout the implementation, key aspects of CSE were encouraged and monitored, including age appropriateness, gender sensitive content and non-judgmental information delivery. Delivery of the intervention occurred between August 2016 and April 2017.

### Evaluation of the intervention

#### Study design

We conducted a mixed methods study to evaluate the effectiveness of this intervention, employing a cluster randomized trial (NCT03669913) as well as post-intervention qualitative interviews and focus group discussions among study participants in the intervention arm. The study assessed changes in sexual health knowledge, sexual behavior, body image, self-esteem and gender equitable norms over a period of 1 year. The study adhered to CONSORT guidelines for reporting of clinical trials [23].

### Quantitative methods

#### Sample size

The cluster randomized trial involved 15 intervention schools and 18 control schools. These were mainly publically funded schools (*n* = 31) with a few private schools (*n* = 2) which is representative of the local context. Sample size estimation was based on simulations in R software. We hypothesized a 10% greater increase in scores for main outcomes between baseline and end-line assessments in the intervention arm compared to the control arm. Taking into account the intra-class correlation calculated from the pilot test among 105 pupils, it was determined that a minimum sample size of 846 would be needed to measure a difference of 10% between groups (reference 50%) with a power of 90%. With an estimated drop-out rate of approximately 20% between baseline and endline assessments, which were 1 year apart, the minimum sample size at baseline was determined to be 1100 pupils in 33 clusters. Within each school, an average of 34 pupils were included in our initial sample.

#### Selection of participating schools and individual study participants

The study was conducted within primary level schools in Mbarara district, found in South Western Uganda. A list of 248 primary schools located in both rural and urban regions was available from district records. We eliminated schools that had no upper classes (primary 5 to 7), and schools that offer unique services, including schools for children with hearing and visual impairments. The names of the rest of the schools were entered into an Excel spreadsheet and a formula was developed to randomly select the required 33 schools for the study. Then an independent statistician randomly assigned each of the 33 schools to the treatment or control arm, using a new Excel formula. Research assistants (RAs) selected the final sample of pupils per school from class registration lists using systematic sampling methods and substituting pupils who were absent on the interview day.

#### Study procedures

To assess the effectiveness of the intervention, we developed a questionnaire (ref: Additional file 1, supplementary material) which was piloted among 105 adolescents. The questionnaire was developed in English and administered in English and in the local language (Runyankole). RAs interviewed students and recorded their responses on paper surveys, which were later entered into a computer database. All RAs were fluent in both languages and administered questionnaires based on each adolescent’s language preference. Additionally, student’s names were not included on the paper survey on which RAs recorded their responses. Instead, we used unique identification numbers in an effort to maintain privacy.

Pupils were interviewed at baseline before the start of the intervention in June and July 2016. After the nine-month intervention was completed, pupils were interviewed again in June and July 2017. Efforts were made to trace pupils who were not available at the day of the interview but could be located within 1 month.

### Outcome measures

#### Sexual and reproductive health knowledge

SRH knowledge was measured based on knowledge of puberty, HIV/STIs and pregnancy prevention. The students were asked questions based on the planned intervention material and were expected to come up with correct answers (as opposed receiving multiple choice questions). This included knowledge on how HIV/AIDS can be acquired, such mother to child transmission, unsterilized instruments or unprotected sex (score 0–4), types of common Sexually Transmitted Infections, such as chlamydia, HIV, gonorrhea and syphilis (score 0–4), knowledge of physical changes during puberty in boys, such as growth of beards, enlargement of genitalia, and acne (score 0–6) and in girls, such as enlargement of breasts, pubic hair and enlargement of hips (score 0–7), and knowledge about ways to prevent pregnancy, such as abstinence and other contraceptive methods (score 0–4). We adapted some of these questions on STI/HIV/Contraception awareness from the WHO illustrative questionnaire for interviews with young people [24]. Due to the age group in question, we also added relevant questions on pubertal changes occurring in boys and girls.

#### Sexual wellbeing and attitudes

Self-esteem scores were estimated using 7 of 10 items of the Rosenberg (1965) self-esteem scale [25]. Body image scores were estimated using 5 of the 6 items of the Body Image States Scale (BISS-6) [26]. Gender equitable norms scores were estimated using 11 items. Six of these items were adapted from the Attitudes towards Women Scale for Adolescents (AWSA) [27] and 5 items were developed to suit the respondents’ age and the Ugandan context. We have described these scales in detail in a previous publication [28].

#### Sexual behavior

Sexual behavior was defined as experience with consensual heterosexual intercourse. Given the political climate around adolescent sexuality and homosexuality in Uganda, we were unable to ask VYAs about their sexuality beyond heterosexual intercourse. Owing to the fact that we were dealing with very young adolescents, interviewers selectively asked questions about sexual activity to only participants who reported that they had been to a private place with a peer of the opposite sex. RAs defined heterosexual sex for pupils as “a boy inserting a penis into the vagina.” Sexual behavior was then classified using a binary yes/no question regarding engagement in sexual activity prior to the study or within the intervention year. Sexual behavior included early sexual onset as well as risky sexual practice (sex without a condom). Given the young age of our study population, those who were already engaging in sex reflected early sexual onset, which we also considered risky sexual behavior.

#### Other variables

We assessed relevant pupil characteristics including age, gender, socio-economic status, orphanhood status and truancy. A socio-economic score was developed based on household water source, distance from water source, household possessions and pupil possessions including shoes and pairs of school uniforms, as we described previously [28]. We also assessed truancy, which was defined as missing school for any reason other than illness. We selected individual and interpersonal variables that could affect the key outcomes among very young adolescents.

#### Quantitative data analysis

Statistical analyses were conducted using Stata® (College Station, Texas, USA). Baseline characteristics at the cluster (school) and individual levels were summarized using group totals and proportions. All outcome variables were analyzed as ordinal categorical variables, with the exception of sexual behavior which was analyzed as a binary variable. Where there were outliers, we grouped the extreme ends of the scales to address skewed data. Specifically, the sexual health knowledge scale was collapsed from 22 categories into 20, gender equitable norms from 30 categories into 24, self-esteem from 18 categories into 14, and body image from 18 categories into 14. Cronbach alpha scores for individual scales ranged from 0.61 to 0.68 at baseline, and from 0.61 to 0.76 at endline. An interaction variable was created using the round variable (baseline vs endline) and study arm variable (intervention vs control). We then used an ordered logistic regression to compare differences in the change from baseline to endline between the intervention and control arms, also known as the “difference in differences” method [22]. We conducted both a univariate analysis and a multiple regression analysis controlling for key covariates, including age, gender, school location (rural vs urban), truancy, and orphanhood. We conducted a second multivariate analysis splitting the groups by gender, to assess whether there was a difference in the treatment effect between boys and girls.

#### Qualitative methods

The qualitative interviews were conducted in a broader context of the study as part of a process evaluation of the CSE intervention [29]. Table 1 is a summary of participant characteristics. Fourteen In-Depth Interviews (IDIs) using an open-ended interview guide were conducted with 4 pupils, 8 teachers and 2 parents. The participants were selected purposively. The pupils and teachers were stratified into two sub-categories; that is the rural and urban schools. The IDI guides comprised open-ended questions which elicited responses related to experiences with CSE, SRH knowledge, SRH behavior and sexual well-being of young adolescents. The interviews were conducted in English for pupils and teachers and Runyankole for parents.

**Table 1:** Characteristics of the qualitative study sample

Study sample	Characteristics	Number of participants
IDIs		
Pupils (*n* = 4)	Female	2
	Male	2
Teachers(*n* = 8)	Male	2
	Female	6
	Urban School	4
	Rural school	4
Parents	Female	1
	Male	1
FGDs		
Pupils (*n* = 2)	Rural school	8
	Urban school	8
Parents (*n* = 1)	Mixed gender	12

We conducted three focus group discussions (FGDs); one with parents and two with pupils comprising 8–12 participants selected purposively. The pupils were identified from within the schools where the CSE intervention was conducted and the schools were stratified into rural and urban settings. The FGDs were conducted in English for the pupils and Runyankole for the parents. The FGDs were conducted to help to establish overall experiences, opinions and attitudes towards the CSE intervention. The FGD guide and IDI guides were pretested to check for accuracy and consistency and to improve validity before the actual data collection began. The IDI and FGD consent forms were translated from English to Runyankole for FGD participants who could not comprehend the English language. Trained research assistants were recruited to conduct the data collection.

#### Qualitative data analysis

The IDIs and FGDs were audio-recorded. The recordings were transcribed verbatim and reviewed in comparison to field notes. Thematic analysis was used for qualitative data analysis. A code list was generated based on the objectives of the study and the outcome variables of the quantitative survey. Coding and analysis were done using Atlas.ti software, where segments from the data were copied and assigned to the generated codes. Texts were coded and clustered along emergent themes in the data and these were later organized according participant’s experiences of the CSE intervention. The qualitative data were presented in narrative form.

#### Ethical considerations

The study was conducted in accordance with the principles defined by the World Health Assembly of 1975 with regard to the ethics principles of research involving human subjects. Ethical approval was received from the Mbarara University Research Ethics Committee (REF MUIRC/7), Uganda National Council of Science and Technology (SS 4045), Ghent University Hospital Ethics Board and registered with clinicaltrials.gov (NCT03669913). We obtained written informed consent from the school head teachers and parents/guardians and assent from pupils prior to data collection. Authorization was obtained from the local ethics board to allow teachers to consent as legal guardians for pupils if a parent was not available at the time of the interview, and efforts were made to inform these parents of their child’s participation. A psychiatrist was available to offer counselling to adolescents whenever this was required. The involvement of the psychiatrist was part of the ethical obligation to address any emerging adverse events such as psychological distress and this did not in any way interfere with the intervention activities. Furthermore the information collected from counselling sessions was not used in the study.

## Results

In this section, we will report the main findings from the impact evaluation using three main outcomes: SRH knowledge, SRH behavior, and sexual wellbeing and attitudes. Each outcome measured quantitatively will be supported by the qualitative findings from interviews and FGDs.

### Characteristics of study participants

**Fig. 1:**
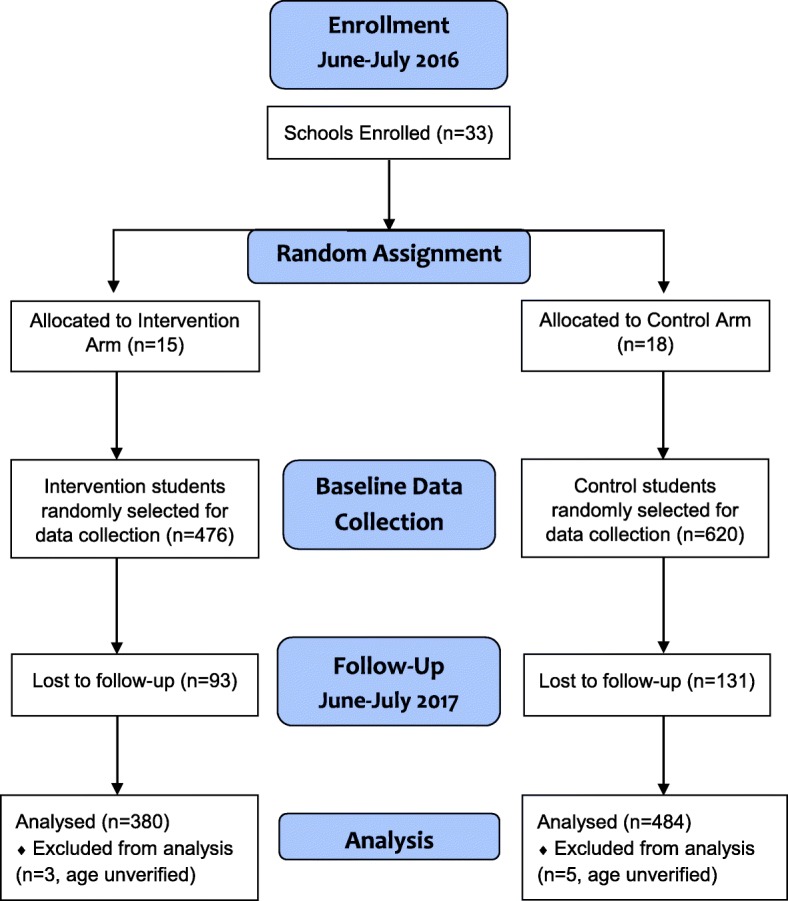
Flow chart describing screening and distribution of participants per study arm

Pre and post intervention surveys were conducted in June and July of 2016 and 2017 among young adolescents in 33 primary schools in Mbarara district, Uganda. A total of 1096 adolescents (476 in intervention and 620 in control schools) were interviewed at baseline and 864 (380 in intervention and 484 in control schools) were interviewed at endline (Fig. 1). The total attrition rate from baseline to endline was 20.6%. The reasons participants were lost to follow up were changing schools (68.5%), dropping out of school (22.5%), getting married (0.9%) and others (8%). The attrition rate was not significantly different between treatment and control groups. Of the 33 participating schools, those from the rural areas represented 13/15 of schools in the intervention arm and 14/18 in the control. Other characteristics of the schools (clusters) are included in Table 2. At endline, participants’ ages ranged from 11 to 15 years.

**Table 2:** Description of participating schools (clusters)

School characteristics	Intervention *n* = 15	Control *n* = 18
School location		
Rural	13	14
Urban	2	4
School care		
Day school	14	12
Day and boarding	1	6
School sponsor		
Government	15	16
Private	0	2
School religious background		
Anglican	11	7
Catholic	3	8
Moslem	1	2
Pentecostal	0	1

The mean age, gender distribution, and orphanhood status were similar among intervention and control arms. However, there were significant differences in socio-economic level, religion and school location (rural vs urban) between the intervention and control arms at baseline (Table 3).

**Table 3:** Baseline social and demographic characteristics for the intervention and control groups

Characteristics	Baseline		
	Total n (%)	Intervention	control
Age (mean, SD)	12.1 (1.13)	12.2 (1.03)	12.1 (1.20)
Gender			
Male	357 (41.3)	147 (38.7)	210 (43.4)
Female	507 (58.7)	233 (61.3)	274 (56.6)
Socio-economic level^a^			
Low	272 (31.8)	116 (30.8)	156 (32.6)
Medium	394 (46.0)	194 (51.4)	200 (41.8)
High	190 (22.2)	67 (17.8)	123 (25.6)*
Parent alive/not			
One/both dead	147 (17.0)	72 (19.0)	75 (15.5)
Both alive	717 (83.0)	308 (81.0)	409 (84.5)
Education level			
Primary 5	438 (50.7)	198 (52.1)	240 (49.6)
Primary 6	426 (49.3)	182 (47.9)	244 (50.4)
School location			
Rural	719 (83.2)	337 (88.7)	382 (78.9)**
Urban	145 (16.8)	43 (11.3)	102 (21.1)

^a^Socio-economic status assessed as a sum of the score for best water source, housing possessions and pupil possessions such as shoes or school uniform pairs with a possible score range of 1–25, median of 8.1. Scores were categorized as “Low” if scores were 1–5,“medium” if scores were 6–10, and “high” if scores were 11 or higher

*P = < 0.01 **P = < 0.001

### Effectiveness of the intervention

#### Sexual health knowledge

The median score for SRH knowledge changed from 8 to 12 in the intervention arm as compared to 8 to 11 in the control arm (Table 4). On further analysis, Sexual Health Knowledge scores improved significantly more among the treatment group compared to the control group. At a bivariate level, the treatment group had 2.14 (*p* < 0.001) times the odds of having greater score improvements than the control group. At multivariate analysis when adjusting for relevant covariates, the treatment group was 2.17 (p < 0.001) times as likely to have greater improvements in SRH knowledge scores (Table 5). When stratifying by gender, we found that the treatment effect was significant in both boys and girls.

**Table 4:** Comparison of sexual health outcomes among young adolescents (unadjusted)

	Intervention		Control	
	Baseline	Endline	Baseline	Endline
Sexual Health Knowledge^a^, *Median (Mean)*	8 (8.1)	12 (12.4)	8 (8.4)	11 (11.1)
Gender equitable norm^b^ *Median (Mean)*	29 (28.2)	29 (28.8)	28 (28.1)	28 (28.3)
Self-esteem score^c^ *Median (Mean)*	24 (23.9)	24 (23.8)	24 (23.6)	24 (23.7)
Body image score^d^ *Median (Mean)*	22 (21.1)	22 (20.9)	22 (21.0)	22 (21.2)
Ever had sex, n (%)	34 (9.0)	46 (12.1)	25 (5.2)	36 (7.4)

^a^Sexual health knowledge was a summation of knowledge on pubertal changes, knowledge on how HIV is acquired, and contraception, with a maximum possible score of 25 (actual score range 1 to 20)

^b^Gender norms were assessed using items from the Attitudes Towards Women Scale and selected context specific gender attitudes on a 4-point likert scale, with a maximum possible score of 44 for 11 items (actual score range 16 to 39)

^c^Self esteem was estimated using a modified version of the Rosenberg scale, including 7 out of 10 items in the original scale, with a maximum possible score of 28 (actual score range 15–28)

^d^Body image score was estimated using a self-reported Body Image State Scale (BISS) with 5 of the original 6 items on a 5-point likert scale with a maximum possible score of 25 (actual range to 12–25)

**Table 5:** Bivariate and multivariate comparison of changes from baseline to endline using ordered logistic regression

Outcome	Bivariate OR (95% CI)	Multivariate AOR (95% CI)
SRH Knowledge	2.14 (1.64–2.79)**	2.18 (1.66–2.86)**
Gender Equitable Norms	1.12 (0.81–1.56)	1.14 (0.80–1.62)
Self Esteem	0.94 (0.65–1.35)	0.93 (0.64–1.33)
Body Image	0.85 (0.56–1.29)	0.84 (0.54–1.31)
Sexual Activity^a^	0.76 (0.33–1.75)	0.76 (0.32–1.80)

^a^Used logistic regression

**P = < 0.001

#### Sexual wellbeing and attitudes

The median scores for gender equitable norms, body image and self-esteem did not differ between intervention and control groups at baseline or at endline (Table 4). We did not find any statistically significant differences in changes of gender equitable norms, body image, or self-esteem scores between the intervention and control groups (Table 5). This was the case in both the bivariate and multivariate analyses as well as among both genders.

#### Sexual behavior

In the intervention group, 34 (9%) pupils at baseline and 46 (12%) at endline reported that they had ever had sex. In the control group, 25 (5%) pupils at baseline and 36 (7%) at endline reported that they had ever had sex (Table 4). We did not find any statistically significant differences between the intervention and control groups in terms of changes in sexual behavior. This was the case in both the bivariate and multivariate analyses, as well as among both genders.

#### Findings from the qualitative analysis

The qualitative findings reveal that study participants recalled many of the CSE topics they had been exposed to during the intervention. Participants reported that they benefited from the topics related to puberty and what they could expect during that life stage. While some pupils reported prior knowledge of some of these topics in their regular classroom hours, they reported that they had gained new information from the CSE session that changed their beliefs and attitudes around puberty and pregnancy. Some examples include:

*“…I didn’t know that at 12 years a boy can make a girl pregnant.” (Student, FGD, rural school)*

*“I learnt that even before menstruation you can get pregnant. It was new to me.” (Student, FGD, rural school)*

“*There is when you would get wet dream and you refuse to go to school but now I know it’s normal.*” *(Student, IDI, rural school)*

The participants reported that the CSE lessons helped them understand body changes and maintenance of personal hygiene, and that they felt more comfortable handling these changes during puberty. They were now able to relate with the opposite sex more comfortably; they became more receptive of CSE information without feeling tense or embarrassed and reported being able have open discussions about these pubertal changes:

“*When you haven’t reached to some stages, there are things they teach and you take them for granted because you haven’t experienced anything….There were things they would say and we become shy and refuse to respond like shaving armpits and private parts. When we reached third term, our teachers also taught them to us and there, we became active and responded because we had heard about them before. Now we cannot hide anything.*” (Student, IDI, rural school)

“…*Previously, like a male teacher discussing menstruation would generate murmurs in the classroom…. but now when you speak those words that were previously thought of as taboo, the children do not even blink; they are informed.* “(Teacher, IDI, rural school)

As noted in the quantitative results, there were no significant differences in sexual behaviours between the intervention and control groups, due in part to the fact that in this young age group there were low rates of sexual activity in both the intervention and control arms. However, in IDIs and FDGs, pupils reported that the CSE sessions influenced their intention to delay sexual intercourse until they were older to prevent unwanted pregnancy, HIV and other STIs, and that the sessions had increased their perception of SRH related risks. In their FGD, parents also gave similar narratives of what the pupils had shared with them regarding their experiences during the CSE sessions about the knowledge on SRH risks that they had acquired:

*“…she told me that yesterday they studied about pregnancy. I asked her what pregnancy means, ‘were they telling you to go and conceive?’ So she started telling me that they taught them about the challenges you meet when you get pregnant when you are still a young girl.”* (Parent, FGD, urban)

The participants also revealed that CSE had a positive effect on some of the socio-cognitive determinants of safe sexual behaviour. Self-esteem and decision making were predominantly reported by the participants. Although participants did not detail lived experiences where they applied self-esteem and decision making, the majority gave positive self-reported statements like the following:

*“I enjoyed self-esteem because before I never believed in myself and now I do and I can make my own decisions.”* (Student, FGD, urban school)

Reports on gender and equitable norms were erratic during the pupil’s interviews. Examples of gender equitable statements included*: “pregnancy and reproduction were the responsibility of both boys and girls,”* (Student, FGD, Urban school) suggesting that students came to better understand that boys and girls were both responsible for and affected by unwanted pregnancy. While students made statements suggesting they understood the importance of gender and equity in the context of the CSE lessons, they did not explicitly discuss how the lessons influenced their attitudes around gender.

## Discussion

This study aims to evaluate the effectiveness of a Comprehensive Sexuality Education intervention among very young adolescents in primary schools in Uganda. This study is among few studies in Uganda to evaluate a reproductive health intervention among very young adolescents. It contributes to an important but limited body of knowledge regarding SRH programming for very young adolescents. The quantitative results of this study show that pupils in the intervention group were two times more likely than those in the control group to have significantly improved scores in SRH knowledge at the endline. However, there were no significant differences between intervention and control groups in the changes in scores on body image, self-esteem, or gender equitable norms, and there was minimal reporting on changes related to these topics in the qualitative data. Although there were no differences quantitatively in sexual behavior, the qualitative data suggested changes in other behaviors related to SRH, such as behavioral intentions and increased perception of SRH related risks.

The finding that CSE interventions can lead to immediate improvements in SRH knowledge has been similarly reported in several other related interventions [13, 30]. However, almost all of these studies focus on older adolescents. Our study highlights the fact that even very young adolescents have great improvements in knowledge following a CSE intervention. This change is important in this context because Uganda’s young people lack comprehensive SRH knowledge, which has been reported in the DHS [6]. Although knowledge does not equate to behavior change, it is a necessary prerequisite. This is important given the young age of these students, who are just beginning to be exposed to SRH risks.

A higher percentage of pupils in the intervention group were sexually active at baseline, which could relate to statistically significant differences in socioeconomic level (*p* < 0.01), urban/rural distribution (*p* < 0.001), or other individual- or school-level factors. Although other studies have found that CSE can reduce risky sexual behaviors [13, 30], our study found no significant differences in sexual behavior between the treatment and control groups. This is likely due in part to the fact that sexual behavior was uncommon among this relatively young group to begin with; only 9.0% of the intervention group and 5.2% of the control group had ever had sex at the beginning of the intervention. Common SRH outcome measures such as sexual initiation, number of sexual partners and condom use that may apply to older adolescents were found less useful when applied to young adolescents in this study who were mostly sexually naïve. Because sexual activity is not very common, it may have been valuable to explore other behaviors related to sexual and reproductive health that are more relevant to this age group. It is important to note that CSE did not lead to more respondents in the intervention group than control group becoming sexually active at the end of the intervention. This finding is important for policy and programming purposes to improve uptake of CSE among decision makers who believe that addressing adolescent sexuality will lead to more young people engaging in sexual activity. The qualitative results highlight age appropriate information regarding benefits of CSE, such as dispelling fears and anxiety in dealing with puberty changes, recognition of SRH risks and how they can be avoided. This study may have benefitted from exploring other sexual behaviors more relevant to this age group, such as precursors to sex, which some similar impact evaluations among this age group have explored [31]. Finally, because pupils were interviewed right after completion of the intervention, long-term behavioral changes might not yet be present and could not be ascertained.

Additionally, while most of these pupils were not sexually active, in focus group discussions and interviews, several pupils discussed behavior intentions. Pupils reported on their intentions to avoid early pregnancy and STIs and to avoid situations that may lead to sexual violence. Even though many of these adolescents had not yet been faced with these decisions, their intentions and skills in future decision making may have changed. Although behavior intention does not necessarily lead to direct behavior change, a meta-analysis of 47 RCTs of behavior change found that medium to large changes in behavior intention do lead to small to medium changes in behavior [32]. Our qualitative data suggest that SRH research among this young age group may provide more meaningful results if it examines behavior intention, and other behaviors related the SRH that are more relevant in this young age group.

This study evaluated dimensions of sexual wellbeing, including gender equitable norms, self-esteem and body image, in order to assess the positive aspects of CSE, as opposed to exclusively assessing risk behaviors. This is one of the first studies to evaluate a CSE intervention in Uganda using these measures, especially among this age group. These measures have been advocated for by experts including the WHO and the European Sexuality Education Expert group, who believe that data on sexual wellbeing can provide important information on the impact of these programs that is often overlooked. However, we did not find any differences between the intervention and control groups, and in fact, there were almost no changes between baseline and endline among either group. This may be attributed to the short interval between the intervention and evaluation, as skill and attitudes take a longer time to change. Also, notably as we described earlier, the initial scores per group for self-esteem and body image were very high [28], leaving limited room for significant improvement. It might be valuable to follow up with these pupils as they get older, when self-esteem and body image tend to vary more [33]. Additionally, measures should be developed that are more tailored to this age group.

This study had several limitations, including a short time interval between the intervention and evaluation. During the study implementation the political environment for sexuality education was prohibitive and essentially a ban was instituted on non-research related dissemination of sexuality education in schools [34]. Although we were able to continue our programming, this prohibitive environment could have affected delivery and/or uptake of the information. Furthermore, the fact that the study population was mostly sexually naïve made it difficult to assess changes in sexual behavior using common measures, such as condom use. We were able to overcome this by establishing qualitative data on applicable behavioral intentions and reporting, but did not measure these alternative outcomes quantitatively. Finally, we did not collect qualitative data among the control students, and are therefore unable to compare qualitative feedback between the two groups.

## Conclusion

This study provides insights regarding the value of implementing SRH education among very young adolescents. It was found that even among these very young adolescents, this intervention led to significant improvements in knowledge. Although knowledge is not sufficient to drive behavior change, it is an important prerequisite for behavior change to occur as adolescents get older. Although we saw no quantitative differences between the intervention and control arms in sexual behavior, and very little sexual behavior overall, the qualitative data suggested changes in behavior intention that could decrease risky sexual behaviors as adolescents get older. More so, it was noted that CSE did not increase sexual activity in the intervention group, which has been a concern among some policy makers in Uganda. This study contributes to the limited body of knowledge on CSE interventions among very young adolescents in Uganda, highlighting the subtle but important advantages of starting this kind of programming at a young age.

## Supplementary information

**Additional file 1.** Interview guide for the study.

## Data Availability

The datasets analysed during the current study are available from the corresponding author on reasonable request.

## Abbreviations

CSE: Comprehensive Sexuality Education
FGD: Focus Group Discussion
IDI: In Depth Interview
RA: Research Assistant
SRH: Sexual and Reproductive Health
SSA: Sub Saharan Africa
VYA: Very Young Adolescent
