# Supplementary material for: Evaluation of a school based comprehensive sexuality education program among very young adolescents in rural Uganda
Source: BMC Public Health. 2019 Oct 28;19:1393. doi: 10.1186/s12889-019-7805-y (PMC6819440; doi:10.1186/s12889-019-7805-y)
Supplement: Supplementary file 1 — Additional file 1. Interview guide for the study. [file 12889_2019_7805_MOESM1_ESM.docx]

**Survey Questionnaire for pupils (Primary 5 to Primary 7**

**Age range – 10 to 14 years)**

Section **A: School characteristics**

*Please fill this page by interviewing any member of the school authority; please ensure the content on this page is uniform for each school cluster*

School ID |__|__|__| Date of Interview |__|__| |__|__| |__|__|__|__|

Day Month Year

| 1. Religious foundation of the school |
| --- |
| Non faith based 1 |
| Catholic 2 |
| Anglican 3 |
| Moslem 4 |
| Born Again Christian 5  Other specify____________ 6 |
| 1. Sponsorship for the school |
| Government aided 1 |
| Privately owned 2 |
| Other Specify 3-------------------- |
| 1. School admission |
| Boys and girls 1 |
| Boys only 2 |
| Girls only 3 |
| 1. School location |
| Rural 1 |
| Urban 2 |
| 1. School care |
| Only day 1 |
| Only Boarding 2 |
| Day and boarding 3 |
| 1. School SRH services (indicate Yes 1 No 2) |
| School nurse \|__\| |
| Senior woman\|__\|  Senior man \|__\|  Youth corner\|__\|  Peer education clubs\|__\|  Other services specify_______________ |

**Section B Social and Demographic Characteristics**

*Please crosscheck the child’s age before administering the questionnaire and ensure they lie in the age group of 10-14 yrs.*

| **No.** | **Question** | **Coding categories** | **Skip to** | |
| --- | --- | --- | --- | --- |
| **1.** | **Demographic Characteristics** | | | |
| 1.1 | What day, month and year were you born? | \|__\|__\|/ \|__\|__\| /\|__\|__\|__\|__\|  Don’t Know 99 \|__\| | |  |
| 1.2 | How old were you on your last birth day? | Years \|__\|__\| | |  |
| 1.3 | Which class are you in? | P5 Class 1  P6 Class 2  P7 class 3 | |  |
| 1.4 | Are you a boy or girl?  (*observe for gender*) | Boy 1  Girl 2 | |  |
| 1.5 | What is your religion? | None 1  Catholic 2  Anglican 3  Moslem 4  Born Again Christian 5  Others specify …….6  No Response 88  Don’t know 99 | | If none or no response skip to 1.8 |
| 1.6 | How much do you like attending religious functions or services? | I like them a lot 1  Not so much 2  Not at all 3  No Response 88  Don’t know 99 | |  |
| 1.7 | In the last 1 month how often did you go to religious service | None 1  **Once or** Twice 2  Three **to four** times 3  More than four times 4  No response 88  Don’t know 99 | |  |

| 1.8 | **Social economic status assessment** | | |
| --- | --- | --- | --- |
| 1.81 | **Water source**  Where does your family get water for home use? | From a tap 1  From a borehole 2  From a dug well 3  From a spring 4  From a rain water tank 5  From a river or stream 6  **Other_________7**  No response 88  Don´t know 99 |  |
| 1.82 | **Where is your water source located?** | Within homestead 1  Outside household 2  No response 88 | If option 1 or 88 skip to 1.84 |
| 1.83 | **Distance from water source**  How long does it take to go there, get water, and come back? | Less than 30mins 1  30mins to 1hour 2  1hour to 2 hours 3  More than two hours 4  No response 88  Don´t know 99 |  |
| 1.84 | **Household floor characteristics**  Tell me about the floor in the house where you live, what is it made of? | Floor with soil or dung 1  Finished with cement or tiles 2  No response 88  Don´t know 99 |  |
| 1.85 | **Household possessions**  At your home do you have any of the following items?  Indicate 1(if present) and 2(if absent) | Electricity \|__\|  Radio \|__\|  Television \|__\|  Telephone \|__\|  Computer\|__\|  Refrigerator\|__\|  Bicycle\|__\|  Motorcycle\|__\|  Car\|__\|  No response 88  Don´t know 99 |  |
| 1.86 | Do you have school shoes? | Yes 1  No 2  No response 88 |  |
| 1.87 | How many pairs of school uniform do you have? | None 1  1 pair 2  2 pairs 3  More than two pairs 4  No response 88 |  |

| 2. | **Section C: School environment and connectedness**  *Now I am going to ask you about your school* | | |
| --- | --- | --- | --- |
| 2.0 | Are you a day scholar or a boarder | Day Scholar 1  Boarder 2 | If 2, skip to 2.3 |
| 2.1 | How did you come to school today? | Walking 1  Riding a bicycle 2  Hired motocycle3  Hired car or van 4  Dropped by a parent by car 5 Dropped by a parent by motorcycle 6  Dropped by parent on bicycle 7  Others specify …….8  No Response 88  Don’t know 99 |  |
| 2.2 | How long did it take you to move from home to school today? | Less than 30mins 1  30mins to 1hour2  1-2 hours 3  More than two hours 4  No Response 88  Don’t know 99 |  |
| 2.3 | Did you miss going to school in the last 1 month? | Yes 1  No 2  No Response 88  Don’t know 99 | If no skip to 2.6 |
| 2.4 | During the last 1 month how many days did you miss school or classes without permission | 1 or 2 days 1  3 to 5 days2  6 to 9 days 3  10 or more days 4  No Response 88  Don’t know 99 |  |
| 2.5 | If Yes 2.3, what do you say is the **main** reason why you missed school? | I did not have school fees 1  I was sick2  My parent/guardian was sick 3  I was lazy to walk the long distance 4  I had my menstrual period 5  I did not have scholastic materials 6  I performed badly at school 7  I went to work to earn money 8  I was banking fees before reporting to school 9  To help with work at home 10  Other reason, please specify--------  No Response 88  Don’t know 99 | **Choose only one** |

| 2.6 | Compared to others in your class how do you rate your performance in your exams? | I am the best1  I am above average 2  I am an average student 3  I am below average 4  No Response 88  Don’t know 99 |  |
| --- | --- | --- | --- |
| 2.7 | To what extent do you like your school? (*give the child options)* | I like my school alot 1  Not so much 2  Not at all 3  No Response 88  Don’t know 99 |  |
| 2.8 | Teachers in my school like me  (To what extent do you agree with this statement) | I agree a lot 1  I Agree a little 2  I Disagree 3  I completely Disagree 4  No Response 88  Don’t know 99 |  |
| 2.9 | If I have a problem at school there is a teacher I can easily talk to | I agree a lot 1  I Agree a little 2  I disagree 3  I completely Disagree 4  No Response 88  Don’t know 99 |  |
| 2.10 | While at school, have you been abused or teased in any way? | **Yes 1**  **No 2** | **If No skip to 3** |
| 2.11 | Describe the abuse  (indicate 1 for yes and 2 for No) | I was kicked, pushed or locked indoors \|__\|  I was made fun of because of my appearance \|__\|  I was made fun of because of my religion\|__\|  I was left out of activities on purpose or ignored\|__\|  Someone made faces at me or other body gesture**\|__\|**  I was bullied in some other way specify_______\|__\|  No response 88  Don’t know 99 |  |
| 2.12 | If yes, who has been responsible for such acts | Peers 1  My teachers 2  Teachers and peers 3  Other specify________ |  |

| **Section D Family Structure and relationships** | | | | | |
| --- | --- | --- | --- | --- | --- |
| 3 | Now I am going to ask you about your family | |  | |  |
| 3.1 | Are your parents alive?  *Choose one option* | | Both mother and father alive 1  Only mother alive 2  Only father alive3  Both mother and father dead 4  No Response 88  Don’t know 99 | |  |
| 3.2 | At home who do you live with?  (indicate 1 yes 2 for No) | | Mother (*indicate 1 or2*) \|__\|  Father (*indicate1 or 2*)\|__\|  Sister (*indicate 1 or 2*)\|__\|  number of sisters(*specify number*)\|__\|  brother( *indicate 1 or 2*)\|__\|  number of brothers(*insert number*)\|__\|  Aunt( *indicate 1 or 2*)\|__\|  Uncle( *indicate 1 or 2*)\|__\|  Grandmother (*indicate 1 or 2*)\|__\|  Grandfather(*indicate 1 or 2*)\|__\|  House help (*indicate 1 or 2*) \|__\|  Non biological parent (*indicate 1 or 2*)\|__\|  **Other at home___________**  No Response 88  Don’t know 99 | | *Multiple response* |
| 3.3 | While at home who do you spend most of your time with?  (indicate 1 yes 2 for No) | | Mother \|__\|  Father\|__\|  Older sister\|__\|  Older brother\|__\|  Aunt\|__\|  Uncle\|__\|  Grandmother\|__\|  Grandfather\|__\|  House help \|__\|  Non biological parent\|__\|  **Other ____________**  No Response 88  Don’t know 99 | | Indicate one yes |
| 3.4 | How do you find talking with your mother about things that are important to you?  (*First let the child describe the important things in his/her life ..)* | | Very easy 1  Easy 2  Difficult 3  Very difficult 4  No Response 88  Don’t know 99 | |  |
| 3.5 | Have you ever discussed boy-girl relationships and love stories with your mother? | | Yes 1  No 2  No Response 88  Don’t know 99 | |  |
| 3.6 | | How do you find talking about boy girl relationships and love stories with your mother? | Very easy 1  Easy 2  Difficult 3  Very difficult 4  No Response 88  Don’t know 99 |  | |
| 3.7 | | How do you find talking about important things to you with your father? | Very easy 1  Easy 2  Difficult 3  Very difficult 4  No Response 88  Don’t know 99 |  | |
| 3.8 | | Have you ever discussed boy-girl relationships and love stories with your father? | Yes 1  No 2  No Response 88  Don’t know 99 |  | |
| 3.9 | | How do you find talking about boy girl relationships and love stories with your father? | Very easy 1  Easy 2  Difficult 3  Very difficult 4  No Response 88  Don’t know 99 |  | |
| 3.10 | | Apart from your parents, which of the following people in your family do you find most easily to talk to about important things in your life? | None 0  Older sister 1  Older brother 2  Aunt 3  Uncle 4  Grandmother 5  Grandfather 6  House maid 7  Other__________  No Response 88  Don’t know 99 |  | |
| 3.11 | | Apart from your parents which of the following people in your family do you find most easily to talk to about, love and boy girl relationships? | None 0  Older sister 1  Older brother 2  Aunt 3  Uncle 4  Grandmother 5  Grandfather 6  House maid 7  Other____________________  No Response 88  Don’t know 99 |  | |

| **4.** | **Section E: Adolescents’ SRH (puberty, relationships, sex, contraception and STIs) knowledge and Behaviors** | | |
| --- | --- | --- | --- |
| 4.1 | **Puberty: The questions below talk about changes that occur in your body as you grow up. The questions may make you uncomfortable, please let me know how you feel**  . Be free not to respond when you are uncomfortable | | |
| 4.11 | **Both boys and girls**: You have been provided with a body chart for girls. Please indicate for me where the body changes occur as one grows into an adult woman  *Provide body chart for girls for the respondent to shade the body changes and for the RA to indicate 1 Yes 2 No* | Increase in height and weight\|__\|  Acne(pimples) \|__\|  Body odour **\|__\|**  Growth of pubic hair\|__\|  Growth of axillary hair (under arms) \|__\|  Breast development\|__\|  Widening of hips\|__\|  Softening of the voice\|__\|  Feels attracted to boys**\|__\|**  Menstruation\|__\|  Others specify ………. 6  No Response 88  Don’t know 99 |  |
| 4.12 | **Girls**: On the given chart below please indicate what you think is your stage of growing up  *Show Tanner stage chart for girls* | Tanner stage 1  Tanner stage 2  Tanner stage 3  Tanner stage 4  Tanner stage 5 |  |
| 4.13 | **Girls:** Have you started experiencing your periods(menstruation)  (indicate 1 yes and 2 No) | Yes 1  No 2  No Response 88  Don’t know 99 |  |
| 4.14 | **Both boys and girls**: You have been provided with a body chart for boys. Please indicate for me where on the body changes occur as one grows into an adult man  *Provide body chart for boys for the respondent to shade the body changes and for the RA to indicate 1 Yes 2 No* | Increase in height and weight \|__\|  Shoulder become broader \|__\|  Acne(pimples) \|__\|  Body odour **\|__\|**  Growth of pubic hair \|__\|  Growth of hair under arms \|__\|  Growth of facial hair(beards) \|__\|  Increased size of genitalia (penis and testicles) \|__\|  Produces semen/Wet dreams \|__\|  Attraction to the opposite sex \|__\|  Voice becomes deeper **\|__\|**  Others specify ………. 7  No Response 88  Don’t know 99 | If it’s girl go to  4.17 after this question |
| 4.15 | **Boys**: On the given chart below please indicate the stage you are regarding growing up  *Show Tanner stage chart for boys* | Tanner stage 1  Tanner stage 2  Tanner stage 3  Tanner stage 4  Tanner stage 5 | Tanner score boys |
| 4.16 | Ask the boy**:** Have you ever woken up to find some little wet patch in your underwear or on your bed?  (indicate 1 yes and 2 No) | **Yes 1**  **No 2**  No Response 88  Don’t know 99 |  |
| 4.17 | Both boys and girls: I look forward to be an adult man/woman  *How much do you agree?* | I agree a lot 1  I Agree a little 2  I Disagree 3  I completely Disagree 4  No Response 88  Don’t know 99 |  |
| 4.18 | Both boys and girls: I think the changes occurring in my body to make an adult person make me feel good  *How much do you agree*? | I agree a lot 1  I Agree a little 2  I Disagree 3  I completely Disagree 4  No Response 88  Don’t know 99 |  |
| 4.19 | Both boys and girls: I am scared of the changes happening(or about to happen) in my body as I grow up  *How much do you agree*? | I agree a lot 1  I Agree a little 2  I Disagree 3  I completely Disagree 4  No Response 88  Don’t know 99 |  |

| 4.2 | **Relationships**  **In this section we shall talk about friendships and what you do with your friends**  **The things that we discuss here will be kept secret between you and me**  **If anything I ask makes you uncomfortable, feel free not to respond** | | |
| --- | --- | --- | --- |
| 4.21 | How many best friends do you have? | None 1  1 friend 2  2 Friends 3  3 or more friends 4 |  |
| 4.22 | Are those best friends’ girls, boys or both? | Only girls 1  Only boys 2  Both Boys and girls 3 |  |
| 4.23 | Have you ever been alone with a girl or boyfriend in a secret/private place? | Yes 1  No 2  No response 88 | If no go to page 14 |

*Instructions to data collectors*

- *If the respondent indicates he has been in a secluded or secret place alone with boyfriend or girlfriend continue from 4.3 to 4.55.*
- *NB: If the course of the interview you suspect this child is sexually active administer the questionnaire for sexually active 4.3 to 4.55*
- *If the respondent indicates he has never been alone in a secret place with a boy-friend or girlfriend proceed to 4.56,*

| 4.3 | **Sexual behavior (only ask if they have ever been alone in a secret place in 4.26)**  **FOR ONLY SUSPECTED SEXUALLY ACTIVE PUPILS PAGE-1** | | |
| --- | --- | --- | --- |
|  | Let’s now talk about what happened between you and your girl or boyfriend in that secret place~~?~~ | | |
| 4.31 | Did you ever kiss on the lips? | Yes 1  No 2  No Response 88  Don’t know 99 |  |
| 4.32 | Did **you** ever put your hand on your friend’s private parts? | Yes 1  No 2  No Response 88  Don’t know 99 |  |
| 4.33 | If yes in 4.32, mention the part(s) touched?  *Indicate Yes 1 or No 2* | Vagina\|__\|  Breast\|__\|  Buttock\|__\|  Penis\|__\|  Other_______ |  |
| 4.34 | Did your friend ever put his or her hand on your private parts | Yes 1  No 2  No Response 88  Don’t know 99 |  |
| 4.35 | If yes in 4.34, mention the part(s) touched?  *Indicate Yes 1 or No 2* | Vagina\|__\|  Breast\|__\|  Buttock\|__\|  Penis\|__\|  Other_______ |  |
| 4.36 | Have you ever had sexual intercourse (meaning a boy inserting a penis into the vagina)? | Yes 1  No 2  No Response 88  Don’t know 99 | **If no Skip to questions on sexually inactive adolescents** |
| 4.37 | What age were you when you first had sex? | Years \|__\|__\| |  |
| 4.38 | With whom did you have the first sexual encounter | A friend 1  A relative 2  A visitor 3  Another family member 4  Someone I don’t know 5  Other_________ |  |
| 4.39 | At the time you had sex for the first time. Would you say you were both willing? | Yes 1  No 2  Unsure 3  No Response 88  Don’t know 99 |  |
| 4.40 | Did you use anything to avoid pregnancy the last time you had sex | Yes 1  No 2  No Response 88  Don’t know 99 |  |

**FOR ONLY SUSPECTED SEXUALLY ACTIVE PUPILS PAGE-2**

| 4.41 | If no to 4.40 why  *Did not use anything to stop pregnancy* | I was shy\|__\|  I did not how to avoid pregnancy\|__\|  I had no money to buy condoms\|__\|  Other_________  No Response 88  Don’t know 99 |  |
| --- | --- | --- | --- |
| 4.42 | If yes what method did you use  *Method to stop pregnancy* | Condom 1  Pill 2  Injection 3  Withdrawal 4  Moon beads 5  Other___________ |  |
| 4.43 | Did you use anything to avoid HIV or other Sexually Transmitted diseases the last time you had sex | Yes 1  No 2  No Response 88  Don’t know 99 |  |
| 4.44 | If no to 4.43  *Did not use anything to stop HIV or STI* | I was shy\|__\|  I did not how to avoid STI/HIV\|__\|  I had no money to buy condoms\|__\|  Other________  No Response 88  Don’t know 99 |  |
| 4.45 | If yes what method did you use  *Method to prevent HIV* | Condom 1  Other ______  No Response 88  Don’t know 99 |  |
| 4.46 | Did you regret having sex the last time you did | Yes 1  No 2  No Response 88  Don’t know 99 |  |
| 4.47 | If yes give reason | It is a sin 1  I was not ready 2  It was painful 3  I was lied to 4  I did not like my partner 5  other |  |

|  | | Indicate to what extent you agree with the statements below  **FOR ONLY SUSPECTED SEXUALLY ACTIVE PUPILS PAGE-3** | | | |
| --- | --- | --- | --- | --- | --- |
| 4.48 | | Have you ever received money or gifts in exchange for sex? | | Yes 1  No 2  No Response 88  Don’t know 99 |  |
| 4.49 | | Girls: I am confident I can control being pregnant | | Yes 1  No 2  No Response 88  Don’t know 99 |  |
| 4.50 | | Boys: I am confident I can control making someone pregnant | | Yes 1  No 2  No Response 88  Don’t know 99 |  |
| 4.51 | | I am confident I can insist on using a condom? | | Yes 1  No 2  No Response 88  Don’t know 99 |  |
| 4.52 | Is it OK for young people your age to have sexual relationships at your age? | | I agree a lot 1  I Agree a little 2  Disagree 3  I completely Disagree 4  No Response 88  Don’t know 99 | |  |
| 4.53 | Is it OK for young people your age to have a sexual relationship with more than one partner at a time? | | I agree a lot 1  I Agree a little 2  Disagree 3  I completely Disagree 4  No Response 88  Don’t know 99 | |  |
| 4.54 | Is it OK for young people your age to have a sexual relationship with a person more than 5years older | | I agree a lot 1  I Agree a little 2  Disagree 3  I completely Disagree 4  No Response 88  Don’t know 99 | |  |
| 4.55 | Do you think it is OK to receive money or gifts in exchange for sex? | | I agree a lot 1  I Agree a little 2  Disagree 3  I completely Disagree 4  No Response 88  Don’t know 99 | |  |

**Sexually naïve Adolescent (*only ask if they have never been alone in a secret place in 4.23 or they have never had sex***. Tell the respondent; some of the questions here may make you feel shy or uncomfortable. You are free to answer those that you are comfortable with

| 4.56 | Do you know what sex is? | Yes 1  No 2  No Response 88  Don’t know 99 |  |
| --- | --- | --- | --- |
| 4.57 | According to you, what do you know about sex? | Being male or female 1  Hugging 2  Kissing 3  Sexual intercourse 4  **Other __________5**  Don’t know 99  No response 88 |  |
| 4.58 | Please tell me **one main** reason why you have decided not have sex at your age?  (*Ask if they know about sex? if no describe like a man and woman sleep together to make a baby) ?*  *Regroup*  *Fear*  *Personal choice*  *External factor*  *Opportunity*  *Others* | I don’t feel ready to have sex 1  I think sex before marriage is wrong 2  I am afraid of getting pregnant/getting someone pregnant 3  I am afraid of getting HIV/AIDS or other sexually transmitted disease 4  it is forbidden in my culture to have sex at this age 5  My parents forbid sex before marriage 6  My religion forbids sex before marriage 7  I have not found a right partner 8  I have not found an opportunity yet 9  I fear being taken to prison **10**  other specify _________  No Response 88  Don’t know 99 | **Choose one**  **Option only** |
| 4.59 | Is it OK for a boy and a girl of your age to go alone in a secret or (private) place? | I agree alot 1  Agree 2  Disagree 3  Strongly Disagree 4  No Response 88  Don’t know 99 |  |
| 4.60 | Is it OK for young people your age to have sexual relationships -? | I agree a lot 1  I Agree a little 2  Disagree 3  I completely Disagree 4  No Response 88  Don’t know 99 |  |
| 4.61 | Is it OK for young people your age to have a sexual relationship with more than one partner at a time? | I agree a lot 1  I Agree a little 2  Disagree 3  I completely Disagree 4  No Response 88  Don’t know 99 |  |
| 4.62 | Is it OK for young people your age to have a sexual relationship with a person more than 5years older ? | I agree a lot 1  I Agree a little 2  Disagree 3  I completely Disagree 4  No Response 88  Don’t know 99 |  |
| 4.63 | Do you think it is OK to receive money or gifts in exchange for sex? | I agree a lot 1  I Agree a little 2  Disagree 3  I completely Disagree 4  No Response 88  Don’t know 99 |  |
| 4.64 | Do you feel pressure to have sex? | Yes 1  No 2  No Response 88  Don’t know 99 | If no skip to 4.67 |
| 4.65 | If YES A great deal or a little? | A great deal 1  A little 2  No Response 88  Don’t know 99 |  |
| 4.66 | From whom do you feel pressure? PROBE AND CIRCLE ALL THAT APPLY | Friends 1  Relatives 2  Partner/special friend 3  Others……….4 |  |
| 4.67 | I am confident I can make an informed choice regarding whether or not to have a sexual relationship? | I agree a lot 1  I Agree a little 2  Disagree 3  I completely Disagree 4  **No Response 88**  **Don’t know 99** |  |

**Section F: Sexuality, Health and Well being**

**In this section we talk about pregnancy and diseases that can spread through sexual intercourse. If there is any disease mentioned that makes you feel sad please feel free to let me know and express your fears**

| 5.11 | How do you think people get pregnant ? | Have sexual intercourse 1  Sleep together 2  Other___________  No response 88  Don’t know 99 |  | | |
| --- | --- | --- | --- | --- | --- |
| 5.12 | Are they ways people can avoid getting pregnant  Indicate yes 1 or No 2 | Abstain \|__\|  Avoid intimate relationships\|__\|  Use pills \|__\|  Use condoms \|__\|  Use Injections \|__\|  Use coils \|__\|  Safe days\|__\|  Others specify____________  No response 88  Don’t know 99 | Multiple answers | | |
| 5.13 | Which sexually transmitted diseases do you know?  Indicate Yes 1 or No 2 | HIV/AIDS \|__\|  Syphilis \|__\|  Gonorrhea \|__\|  Chlamydia \|__\|  Others ……….5  No Response 88  Don’t know 99 |  | | |
| **5.2 Knowledge , opinions and attitudes about HIV/AIDS** | | | | | |
| 5.20 | Can you mention some ways through which someone may acquire HIV  Indicate Yes 1 or No 2 on options | Unprotected sex \|__\|  Blood transfusion \|__\|  Injury from sharing sharp objects from infected person \|__\|  From mother to child \|__\|  Not using condoms \|__\|  Other specify______________ | |  | |
| 5.21 | Is it possible for a healthy-looking person to have HIV/AIDS? | Yes 1  No 2  Don’t know 88  No Response 99 | |  |  |
| 5.22 | Can a pregnant woman infected with HIV/AIDS give the virus to her unborn child during pregnancy or delivery? | Yes 1  No 2  Don’t know 88  No Response 99 | |  |  |
| 5.23 | If a teacher was infected with HIV/AIDS, would you feel comfortable if he continue teaching you in class? | Yes 1  No 2  Don’t know 88  No Response 99 | |  |  |

**Section G**: **Adolescents’ Access to SRH Education – knowledge and attitudes**

|  | **School SRH services, access and quality** | | |
| --- | --- | --- | --- |
| 6.1 | While at school is there a **person** where you can ask questions regarding growing up or relationships? | Yes 1  No 2  No Response 88  Don’t know 99 | If no skip to 6.9 |
| 6.2 | If yes mention the **person** | Senior woman/man 1  School nurse 2  Science Teacher 3  Other specify_________ |  |
| 6.3 | While at school is there a **place** where you can ask questions regarding growing up or relationships? | Yes 1  No 2  No Response 88  Don’t know 99 |  |
| 6.4 | If yes mention the **place** | School clinic 1  School counseling room 2  Classroom 3  Mainhall 4  Other specify__________ |  |
| 6.5 | In the last term, have you talked to this person or visited any placeto seek for services regarding growing up or relationships? | Yes 1  No 2  No Response 88 | If no skip to 6.9 |
| 6.6 | While consulting at school  Did you feel comfortable to ask questions? | Yes 1  No 2  No Response 88  Don’t know 99 |  |
| 6.7 | Did you receive an answer to your questions? | Yes 1  No 2  No Response 88  Don’t know 99 |  |
| 6.8 | Did your conversation take place in a separate room without others being present? | Yes 1  No 2  No Response 88  Don’t know 99 |  |
|  | **SRH service at home/community** | | |
| 6.9 | Is there a **place in your community** where young people like you are able to visit to talk and find out about relationships, sex, contraception, sexually transmitted infections and HIV/AIDS? | Yes 1  No 2  No Response 88  Don’t know 99 | If no skip to 6.15 |
| 6.10 | If yes in 6.8,  Please name the place? | Youth reproductive Centre 1  Health facility 2  Family planning clinic 3  Other (name)_______ |  |
| 6.11 | In the last term, have you visited any facility within your home community to find out about relationships, sex, contraception, sexually transmitted infections and HIV/AIDS services? | Yes 1  No 2  No Response 88  Don’t know 99 | If no skip to 6.15 |
| 6.12 | While consulting at the facility or person within your community  Did you feel comfortable to ask questions? | Yes 1  No 2  No Response 88  Don’t know 99 |  |
| 6.13 | Did you receive an answer to your questions? | Yes 1  No 2  No Response 88  Don’t know 99 |  |
| 6.14 | Did your conversation take place in a separate room without others being present? | Yes 1  No 2  No Response 88  Don’t know 99 |  |
| 6.15 | Which person do you find most suited to offer you education on growing up, relationships, sexually transmitted diseases or HIV? | Mother 1  Father 2  Both mother and father 3  Grandparents 4  Aunties 5  Uncles 6  Teachers 7  Health work 8  Peers 9  Older students 10  Other……… 11  No Response 88  Don’t know 99 |  |
| 6.16 | Where would you prefer to receive SRH information? | On assembly 1  In class during lessons 2  At church/religious event 3  In small groups with my friends 4  In written material like a book or brochure 5  Internet 6  Others ……  No Response 88  Don’t know 99 |  |

| **6.20** | **Media and SRH of adolescent**  **Let us talk about media such like radio, TV, phones and internet** | | |
| --- | --- | --- | --- |
| 6.21 | Do you have access to the following  Indicate yes 1 or No 2 | Radio \|__\|  Television \|__\|  Mobile phone \|__\|  Social media(Facebook/whatsapp) \|__\|  Newspapers \|__\|  Tabloids (hello, redpepper) \|__\|  Other specify__________ | If no, go to 6.23 |
| 6.22 | Indicate how long you spend per day on the media (radio, phone or TV) | None 1  1 hour/day 2  2-3 hours/day 3  4-5 hours/day 4  More than 5hours/day 5 |  |
| 6.23 | Have you listened to an education talk or show on TV or radio regarding the following below:  Indicate Yes 1 No 2 | Growing up(e.g menstruation) \|__\|  Boy/girl relationships \|__\|  Use of condoms \|__\|  Pregnancy \|__\|  Sex\|__\|  HIV/AIDS\|__\|  Sexually transmitted diseases\|__\|  Don’t know 99  No response 88 |  |
| 6.24 | Have you watched a movie showing or describing sexual act or content(pornography) | Yes 1  No 2  Don’t know 99  No response 88 | If no skip to 6.26 |
| 6.25 | If yes, how did you access this material | On a TV program at home 1  I watched it on a smart phone 4  I borrowed a blue(sexual content) movie 5  I used internet to access it 6  Other specify__________ |  |
| 6.26 | Have you read a newspaper where they were describing sex act or content  (**local tabloid papers red pepper, hello, onion etc) | Yes 1  No 2  Don’t know 99  No response 88 | If no skip to 6.29 |
| 6.27 | If yes specify the paper indicate, yes 1, No 2 | Redpepper/Hello**\|__\|**  New vision**\|__\|**  Daily monitor**\|__\|**  Orumuri**\|__\|**  Entatsi**\|__\|**  Other specify______ |  |
| 6.28 | If yes, how did you access it | I read the tabloid at home 1  I bought a tabloid paper 2  A friend gave me the paper 3  I accessed it via internet 4  Other_____________ |  |
| 6.29 | Have you watched a movie or TV program with sexual violence e.g rape | Yes 1 No 2 No response 88 Don’t know 99 |  |

| **7** | **Section H: Gender roles and Norms (AWSA score items)**  **This section is about what boys do and girls do regarding daily activities at home, community or school .Ask respondent to give his or her opinion on the statements below** | | |
| --- | --- | --- | --- |
| 7.1 | Ask boy; do you see yourself as a typical boy  Ask girl; do you see yourself as a typical girl? | 1.Yes  2. No  No Response 88  Don’t know 99 | remove |
| 7.2 | In my home responsibilities of a mother are the same as responsibilities of a father?  *(Ask even if they are orphaned they may have father figure or mother figure*) | I agree a lot 1  I Agree a little 2  I Disagree 3  I completely Disagree 4  No Response 88  Don’t know 99 | Reverse score for equity |
| 7.3 | Who do you regard as a head of your family?  *(Ask even if they are orphaned*) | Father 1  Mother 2  Sister 3  Brother 4  Grandparents 5  No Response 88  Don’t know 99 | Off |
| 7.4 | In my home. boys and girls are equally responsible for household chores.  (*Ask even if it’s an only child or with only brothers or only sisters*) | I agree a lot 1  I Agree a little 2  I Disagree 3  I completely Disagree 4  No Response 88  Don’t know 99 | Reverse score |
| 7.5 | If there is a sick person at home, only a girl should stay home to care for the sick one as the boy goes on with school | I agree a lot1  I Agree a little 2  I Disagree 3  I completely Disagree 4  No Response 88  Don’t know 99 | Ok |
| 7.6 | If in a family where there are girls and boys, priority for school fees should be only for the boys | I agree a lot 1  I Agree a little 2  I Disagree 3  I completely Disagree 4  No Response 88  Don’t know 99 | Reverse score |
| 7.7 | It’s OK for boys to have sex before marriage | I agree a lot1  I Agree a little 2  I Disagree 3  I completely Disagree 4  No Response 88  Don’t know 99 | Remove |
| 7.8 | It’s OK for girls to have sex before marriage | I agree a lot1  I Agree a little 2  I Disagree 3  I completely Disagree 4  No Response 88  Don’t know 99 | Remove |
| 7.9 | Girls should be free to go out unaccompanied to meet with friends | I agree a lot 1  I Agree a little2  I Disagree 3  I completely Disagree 4  No Response 88  Don’t know 99 | Reverse score |
| 7.10 | Boys should be free to go out unaccompanied to meet with friends | I agree a lot 1  I Agree a little2  I Disagree 3  I completely Disagree 4  No Response 88  Don’t know 99 | Reverse score |
| 7.11 | In a family, it’s okay for a husband to beat up a wife if she doesn’t perform her duties | I agree a lot 1  I Agree a little 2  I Disagree 3  I completely Disagree 4  No Response 88  Don’t know 99 | Ok |
| 7.12 | In a family important decisions need to be made by a father | I agree a lot 1  I Agree a little 2  I Disagree 3  I completely Disagree 4  No Response 88  Don’t know 99 | Reverse score |
| 7.13 | It’s a father’s major responsibility more than a mother’s to provide financially for the children | I agree a lot 1  I Agree a little 2  I Disagree 3  I completely Disagree 4  No Response 88  Don’t know 99 | Reverse score |
| 7.14 | Girls should be more concerned about becoming better wives in future than seeking important jobs or businesses | I agree a lot 1  I Agree a little 2  I Disagree 3  I completely Disagree 4  No Response 88  Don’t know 99 | ok |
| 7.15 | My mother more than my father should be blamed for my mistakes | I agree a lot 1  I Agree a little 2  I Disagree 3  I completely Disagree 4  No Response 88  Don’t know 99 | Reverse score |
| 7.16 | Boys are better leaders than girls | I agree a lot 1  I Agree a little 2  I Disagree 3  I completely Disagree 4  No Response 88  Don’t know 99 | Reverse score |
| 7.17 | On average boys are more clever than girls | I agree a lot 1  I Agree a little 2  I Disagree 3  I completely Disagree 4  No Response 88  Don’t know 99 | Reverse score |
| 7.18 | It is alright for a girl to play rough  sports like football. | I agree a lot 1  I Agree a little 2  I Disagree 3  I completely Disagree 4  No Response 88  Don’t know 99 | Reverse score |

**Section I: Assessment of adolescents self esteem and body image scores**

| 8 | **Body image assessment(BISS 6- item score)**  **Regarding the shape or size of your body right now, please tell me how much you agree or disagree/** *describe how you feel* | | |
| --- | --- | --- | --- |
| 8.1 | *Regarding your current looks,*  Right now, I feel | Extremely satisfied with my looks 1  Slightly satisfied with my looks 2  Neither satisfied or dissatisfied with my looks 3  Slightly dissatisfied with my looks 4  Extremely dissatisfied with my looks 5 | 5 to 1 |
| 8.2 | *Regarding your body size and shape*  Right now I feel | Extremely satisfied with my body size and shape 1  Slightly satisfied with my body size and shape 2  Neither satisfied or dissatisfied with my body size and shape 3  Slightly dissatisfied with my body size and shape 4  Extremely dissatisfied with my looks 5 | 5 to 1 |
| 8.3 | *Regarding your weight*  Right now I feel | Extremely satisfied with my weight 1  Slightly satisfied with my weight 2  Neither satisfied or dissatisfied with my weight 3  Slightly dissatisfied with my weight 4  Extremely dissatisfied with my weight 5 | 5 to 1 |
| 8.4 | *Regarding your beauty*  Right now I feel | Extremely beautiful/handsome 1  Slightly beautiful/handsome 2  Neither beautiful or ugly 3  Slightly unattractive(ugly) 4  Extremely unattractive(ugly) 5 | 5 to 1 |
| 8 .5 | **Now you are growing and your body is changing, how do you**  **feel about your looks** | Much worse about my looks than I usually feel 1  A little worse about my looks than I usually feel 2  About the same about my looks as usual 3  Slightly dissatisfied with my looks 4  Much better about my looks than I usually feel 5 | eliminated |
| 8.6 | *Compared to your agemates*  Right now I feel that I look | Much better than an average girl/boy 1  Slightly better than an average girl/boy 2  About the same as an average boy/girl 3  Slightly worse than an average boy/girl 4  Much worse than an average girl/boy 5 | 5 to 1 |

| 9,mv | **Self-esteem score assessment(Rosenberg 1965)**  **Here describe how you feel about your life and your abilities** | | |
| --- | --- | --- | --- |
| 9.1 | I feel that I am a person of importance  (*likely to be a head teacher, an MP or president in future*) | I agree a lot 1  I agree a little 2  I disagree 3  I completely disagree 4 | 4 to 1 |
| 9.2 | I can do things just like most of my age mates | I agree a lot 1  I agree a little 2  I disagree 3  I completely disagree 4 | eliminated |
| 9.3 | I feel useless at times. | I agree a lot 1  I agree a little 2  I disagree 3  I completely disagree 4 |  |
| 9.4 | I feel that I am a failure | I agree a lot 1  I agree a little 2  I disagree 3  I completely disagree 4 |  |
| 9.5 | I feel I do not have much to be proud of.  (*in performance, qualities or skills*) | I agree a lot 1  I agree a little 2  I disagree 3  I completely disagree 4 |  |
| 9.6 | I feel that I have a number of good qualities.  (*in performance, qualities or skills*) | I agree a lot 1  I agree a little 2  I disagree 3  I completely disagree 4 | 4 to 1 |
| 9.7 | On the whole, I am satisfied with myself.  (*I don’t wish to be different*) | I agree a lot 1  I agree a little 2  I disagree 3  I completely disagree 4 | 4 to 1 |
| 9.8 | At times I think I am no good at all  (*feeling unloved, of no purpose in life*) | I agree a lot 1  I agree a little 2  I disagree 3  I completely disagree 4 |  |

**Section J: Sexual violence and reporting**

**The questions in this section may concern experiences that have caused you a lot of pain in the past. Be free to share with us and tell us if you need further help or counseling. If you are uncomfortable sharing your experience, we shall not ask further questions**

| 10.1 | Have you experienced any of the following : Yes 1 No 2 | Bullying including sexual jokes \|__\|  Touching in a sexual way(breast, buttock or genitals) \|__\|  Forceful hugging \|__\|  Forceful kissing \|__\|  Forceful Sexual intercourse \|__\|  **Others specify____________** | **If no to all finish here** |
| --- | --- | --- | --- |
| 10.2 | If yes, by who | Classmate \|__\|  School mate \|__\|  Teacher \|__\|  Relative \|__\|  Parent \|__\|  Housemaid \|__\|  Neighbor \|__\|  Stranger \|__\|  Other_____________  Don’t know 99 |  |
| 10.3 | If yes, did you report to anyone | Yes 1  No 2  No response 88 | **If no skip to 10.6** |
| 10.4 | If yes, who did you report to | Mother 1  Father 2  Teacher 3  Health provider 4  Police 5  Other_________ |  |
| 10.5 | If yes, was the offender punished? | Yes 1  No 2  No response 88  Don’t know 99 |  |
| 10.6 | If no reporting was done, give reason | I was afraid 1  I did not know who to report to 2  **other**_________________ |  |

| **Official Use** | | |
| --- | --- | --- |
| **Date** | **Name of person completing questionnaire** | **Signature** |
| **Date** | **Name of monitor verifying completeness/errors** | **Signature** |
| **Date** | **Data entrant 1** | **Signature** |
| **Date** | **Data entrant 2** | **Signature** |
